# Sodium tanshinone IIA sulfonate ameliorates neointima by protecting endothelial progenitor cells in diabetic mice

**DOI:** 10.1186/s12872-023-03485-4

**Published:** 2023-09-11

**Authors:** Yan-Yan Heng, Hui-Juan Shang, Xia-ze Zhang, Wei Wei

**Affiliations:** 1 Department of Nephrology, Heping Hospital Affiliated to Changzhi Medical College, No.110, Yanan Road South, Changzhi, Shanxi China; 2 Department of Foreign Language Teaching, Changzhi Medical College, No.161, Jiefang East Street, Changzhi, Shanxi China; 3 The First Clinical Acadamy of Changzhi Medical College, No.161, Jiefang East Street, Changzhi, Shanxi China; 4 Department of Pharmacology, Changzhi Medical College, No.161, Jiefang East Street, Changzhi, 046000 Shanxi China; 5 Department of Phase I Clinical Trial Laboratory, National Institute for Clinical Trials of Drugs, Heping Hospital Affiliated to Changzhi Medical College, No.110, South Yan’an Road, Changzhi, 046000 Shanxi China

**Keywords:** Endothelial progenitor cell, Sodium tanshinone IIA sulfonate, NLRP3 inflammasome, Catalase, Neointima

## Abstract

**Background:**

Endothelial progenitor cells (EPCs) transplantation is one of the effective therapies for neointima associated with endothelial injury. Diabetes impairs the function of EPCs and cumbers neointima prevention of EPC transplantation with an ambiguous mechanism. Sodium Tanshinone IIA Sulfonate (STS) is an endothelium-protective drug but whether STS protects EPCs in diabetes is still unknown.

**Methods:**

EPCs were treated with High Glucose (HG), STS, and Nucleotide-binding Domain-(NOD) like Receptor 3 (NLRP3), caspase-1, the Receptor of Advanced Glycation End products (AGEs) (RAGE) inhibitors, Thioredoxin-Interacting Protein (TXNIP) siRNA, and EPC proliferation, differentiation functions, and senescence were detected. The treated EPCs were transplanted into db/db mice with the wire-injured Common Carotid Artery (CCA), and the CD31 expression and neointima were detected in the CCA inner wall.

**Results:**

We found that STS inhibited HG-induced expression of NLRP3, the production of active caspase-1 (p20) and mature IL-1β, the expression of catalase (CAT) cleavage, γ-H2AX, and p21 in EPCs. STS restored the expression of Ki67, CD31 and von Willebrand Factor (vWF) in EPCs; AGEs were found in the HG-treated EPCs supernatant, and RAGE blocking inhibited the expression of TXNIP and the production of p20, which was mimicked by STS. STS recovered the expression of CD31 in the wire-injured CCA inner wall and the prevention of neointima in diabetic mice with EPCs transplantation.

**Conclusion:**

STS inhibits the aggravated neointima hyperplasia by protecting the proliferation and differentiation functions of EPC and inhibiting EPC senescence in diabetic mice. The mechanism is related to the preservation of CAT activity by inhibiting the RAGE-TXNIP-NLRP3 inflammasome pathway.

**Supplementary Information:**

The online version contains supplementary material available at 10.1186/s12872-023-03485-4.

## Introduction

Diabetes aggravates neointima hyperplasia which is the basic pathological change of stenosis and restenosis in coronary artery bypass grafting, percutaneous coronary intervention, and atherosclerosis [1–4]. The Endothelial Progenitor Cell (EPC) is the stem cell of renewing endothelium, which is considered as one of the effective therapies for neointima [5, 6]. Diabetes impairs the function of EPC endothelial repair and induces EPC apoptosis and senescence [7, 8], but the mechanism of High Glucose (HG)-induced EPC dysfunction remains unclear.

Sodium Tanshinone IIA Sulfonate (STS) is the water-soluble modification of tanshinone IIA, which is an extract of the root of the traditional Chinese medicine *Salvia miltiorrhiza* [9]. Because of its anti-inflammatory, anti-oxidative, and anti-apoptotic effects, STS is a widely-recognized clinical drug for treating cardiovascular diseases [10–12]. Recent studies have shown that by inhibiting tumor necrosis factor-α, tanshinone IIA down-regulates the expression of Vascular Cell Adhesion Molecule 1 (VCAM-1) and Intercellular Adhesion Molecule-1 (ICAM-1) in EPC and improves the damaged cell functions of EPC, such as proliferation, migration, adhesion, and tubular formation, which provides a potential pharmacological effect on preventing atherosclerosis [13, 14]. However, the mechanism of STS protecting the function of EPC in HG conditions remains unclear.

This study aims to observe the STS protective effects on EPC in neointima hyperplasia aggravated by diabetes, and reveal the mechanism of alleviating the dysfunctions of EPC by STS treatment in HG.

## Materials and methods

### Isolation, culture, and characterization of mouse bone marrow-derived EPCs

EPCs were isolated from mouse bone marrow according to the previous study [15]. Density gradient centrifuged the bone marrow medulla with a Human peripheral blood lymphocyte isolation fluid (Ficoll Plus 1.077, Solarbio, P4350, Beijing, China) to obtain the bone marrow Mononuclear Cells (MNCs). MNCs were plated in fibronectin (Solarbio, F8180, Beijing, China) coated cell culture bottle with a ventilation filter and maintained in Endothelial Growth Medium-2 (EGM-2, Lonza, CC-4176, USA) with 20% fetal bovine serum (FBS, EPHRAIM, 26–500-FBS China) in a cell incubator with 37℃ and 5% CO_2_. EPCs were treated with HG (35 mM), Lipopolysaccharide (LPS, 1 μg/ml, Sigma-Aldrich, USA) + Adenosine Triphosphate (ATP, 5 mM, ALADDIN, shanghai, China) for 4 h, and STS (100 μM, S107694, ALADDIN, Shanghai, China). EPC characterization is shown in the Supplemental Fig. 1. Cells were double-positive stained by DiI-ac-LDL and FITC-UEA-I (Supplementary Fig. 1a), and CD34, CD133, and VEGFR2 positive (Supplementary Fig. 1b). These characteristics were consistent with the previous descriptions of EPC [16, 17].

**Fig. 1:**
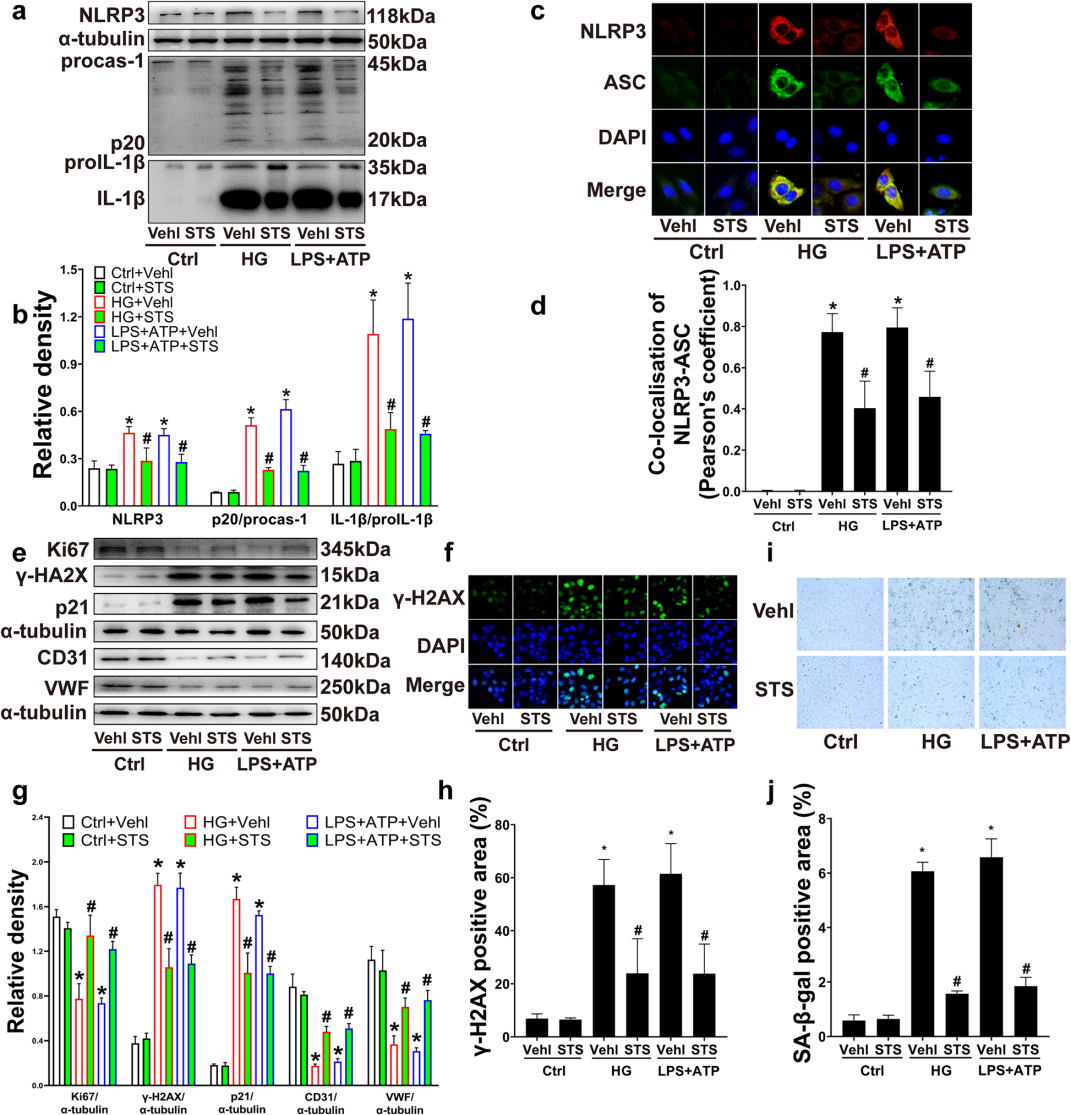
STS inhibited HG-induced EPC NLRP3 inflammasome activation and dysfunctions. **a**, **b** Representative Western blot gel and summarized data show the protein expression of NLRP3, p20, and IL-1β production; **c**, **d** Representative immunofluorescence images (400 ×) and summarized coefficient of colocalization of NLRP3 and ASC; **e**, **f** Representative Western blot gel and summarized data show the protein of Ki67, γ-H2AX, p21, CD31, VWF expression, **g**, **h** Representative immunofluorescence images of γ-H2AX in the nucleus (200 ×) and summarized percent of the γ-H2AX positive area in nucleus total area; **i**, **j** Representative images of SA-β-gal staining (100 ×) and summarized data show the percent of SA-β-gal positive area. *P < 0.05 vs. Control (Ctrl); #*P* < 0.05 vs. HG or LPS + ATP treated group (*n* = 3)

### Animals, wire-injured neointima model, EPCs transplantation

Spontaneously diabetic mice db/db mice and the littermate (Wild-Type, WT) were purchased from the GemPharmatech, Nanjing, China. Mice common carotid artery (CCA) were injured with a flexible wire (0.38 mm diameter) which was inserted from the external carotid artery and passed eight times in CCA to induce neointima hyperplasia. Six hours after the wire-injury operation, mice were treated with STS (gavage 10 mg/kg/day, S107694, Aladdin, Shanghai, China), MCC950 (gavage 20 mg/kg/day, HY-12815, MCE, USA), Z-WEHD-FMK (WEHD, intraperitoneal injection 3 mg/kg/d, A1924, ApexBio Technology, USA), and Azeliragon (TTP488, intraperitoneal injection 4 mg/kg/d HY-50682, MCE, USA), and EPCs (1 × 10^6^) or TXNIP siRNA (sc-44944, VDUP1 siRNA Santa Cruz, USA) pretreated-EPCs were transplanted via tail vein. A portion of the CCAs was harvested in one week, and the ratio of CD31 coverage in the inner vascular wall was detected by immunofluorescence. The remaining CCAs were harvested in the eighth week, and the intima/media was detected by Hematoxylin-Eosin staining (HE).

### Western blot analysis and co-immunoprecipitation

Western blot analysis was performed as described previously [18]. Briefly, cell total protein and membrane protein samples were extracted by using Membrane and Cytosol Protein Extraction Kit (Beyotime, P0033, Shanghai, China), and nuclear protein samples were extracted by using Nuclear and Cytoplasmic Protein Extraction Kit (Beyotime, P0027, Shanghai, China). Protein concentration was quantified by using a BCA Protein Assay Kit (Keygentec, KGP903, Nanjing, China). According to the quantification, protein samples were adjusted to equal with 5 × loading buffer (Beyotime, P0015, Shanghai, China), and samples were boiled for 5 min at 95°C. Equal amounts of cell lysate protein were resolved by SDS-PAGE using 5% (w/v) stacking and 8%-15% (w/v) separating polyacrylamide gels. The whole piece of separated gel was cut according to the molecular weight of the target protein, then the cut-gels were transferred to 0.45 μm polyvinylidene difluoride membrane (PVDF, Millipore, USA), which were then blocked for 1.5 h in 5% (w/v) non-fat milk diluted in Tris-buffered saline (TBS, 100 mM Tris–HCL, pH 7.4) with 0.01% (v/v) Tween-20, and incubated with the primary antibodies overnight at 4°C. The primary antibodies used were rabbit anti-TXNIP (1:500, Beyotime AF8277, Shanghai, China), rabbit anti-NLRP3 (1:1000, Abways Technology, Inc., CY5651, Shanghai, China), rabbit anti-p20 (1:500, Abways Technology, Inc., AY0406 Shanghai, China), rabbit anti-Ki67 (1:500, Wanleibio, WL01384a, Shenyang, China), rabbit anti-IL-1β (1:1000, Wanleibio, WL00891, Shenyang, China), rabbit anti- Histone H2A.X (1:500, Wanleibio, WL00616a, Shenyang, China), rabbit anti-CD31 (1:500, affinity, P16284, Changzhou, China), rabbit anti-vWF (1:1000, affinity, AF3000, Changzhou, China), rabbit anti-pan-cadherin (1:1000, Wanleibio, WL03295, Shenyang, China), rabbit anti-α-tubulin (1:4000, Abways, AB0048, Shanghai, China), rabbit anti-Histone H3 (1:1000, Wanleibio, WL0984a, Shenyang, China). After being washed for three times, the membranes were incubated with goat anti-rabbit IgG (1:5000, Abways Technology, Inc., Shanghai, China) for 1.5 h. The blot was detected by an automatic chemiluminescence/fluorescence image analysis system (5200 Multi, Tanon, Shanghai, China) with electrochemiluminescence (ECL, Tanon, Shanghai, China). Densitometric analysis of the images was performed with Image J software (NIH, Littleton, CO, USA).

#### Co-immunoprecipitation (Co-IP)

According to our report [19]. The total protein of treated EPCs (2 × 10^7^) was extracted by using RIPA Lysis Buffer (Beyotime, P0013B, Shanghai, China). 1 μg Rabbit IgG (Bioworld technology, co, Ltd, Nanjing, China) and 20 μl Protein A + G Agarose (Beyotime, P2055, Shanghai, China) were added to each sample to remove the nonspecific binding. The primary rabbit anti-catalase antibody (Abways, CY6783, Shanghai, China) was added and combined with catalase, and samples were shocked slowly overnight at 4℃. The immunoprecipitation was performed by using 30 μl Protein A + G Agarose for each sample for 2 h, 4℃. After 1000 g centrifugation, the supernatant was discarded, the precipitate was suspended with 1 × SDS loading buffer (Beyotime, P0015A, Shanghai, China), and boiled at 100℃ for 3 min. Samples were separated by SDS-PAGE gel, and CAT-p20 conjunction was detected by rabbit anti-p20 antibody (Wanleibio, WL02996a, Shenyang, China). As same as these steps, the p20-CAT conjunction was detected by the rabbit anti-catalase antibody (Abways, CY6783, Shanghai, China).

### Catalase activity detection

The activity of catalase was detected by in-gel catalase stain [20]. Cells were lysed by RIPA lysis buffer (Beyotime, P0013B, Shanghai, China) with Phenylmethanesulfonyl fluoride (PMSF, Beyotime, ST506, Shanghai, China) on ice, and the extract was centrifuged for 10 min at 4℃ and 10000 g. The supernatant was mixed with non-reducing 4 × SDS loading buffer (50 mM Tris–HCl, pH 6.8, 2% SDS, 10% glycerol, 12.5 mM EDTA, 0.02% bromophenol blue) and electrophoresed on a 10% PAGE gel in electrophoresis buffer (3.025 g tris, 14.4glycine dissolve in 1L deionized water). The gel was washed with deionized water three times and then immersed in 0.003% H_2_O_2_ (2 μl 30% H_2_O_2_ added to 20 ml deionized water) for 10 min and stained with a freshly staining solution (2% potassium ferricyanide and 2% ferric chloride) until the bright band appeared. After washed three times by running water, the gels were photographed by an automatic chemiluminescence/fluorescence image analysis system (5200 Multi, Tanon, Shanghai, China). The α-tubulin detected by western blot was used to normalize the total protein.

### Immunofluorescence analysis

#### Cell experiment

EPCs (5 × 10^4^ cells) were seeded on sterilized coverslips. After treatment, cells were washed three times with PBS (137 mM NaCl, 2.7 mM KCl, 10 mM Na_2_HPO4, 1.8 mM KH_2_PO4) and fixed by 4% paraformaldehyde (PFA, C104188, ALADDIN, Shanghai, China) for 30 min. The cell membrane was perforated by 0.3% Triton X-100 (Biosharp, BS084, Hefei, China) for 15 min, and the cell was blocked with 5% bovine serum albumin (BSA, Solarbio, A8010, Beijing, China) for 1.5 h. The primary antibodies rabbit anti-NLRP3 (1:200, Abways Technology, Inc., CY5651, Shanghai, China), rabbit anti-PYCARD (ASC, 1:100, Abways Technology, Inc., AY0406, Shanghai, China), rabbit anti-CD31 (1:100, affinity, P16284, Changzhou, China), rabbit anti-α-tubulin (1:1000, Abways, AB0048, Shanghai, China), rabbit anti-γ-H2AX (1:100, Proteintech, 10856-1-AP, Wuhan, China) were incubated for 5 h, respectively. After triple washed by PBS, NLRP3, and CD31 were labeled by the second fluorescent antibody Cy3–conjugated Affinipure Goat Anti-Rabbit IgG(H + L) (1:100, Proteintech, SA00009-2, Wuhan, China), and ASC, α-tubulin, γ-H2AX were labeled by the second fluorescent antibody Goat Anti-Rabbit IgG (H + L) Alexa Fluor 488 (1:100, Abways Technology, Inc., AB0141, Shanghai, China) for 2 h, respectively.

#### Tissue experiment

CCAs were cut at 5 μm by a freezing microtome (KEDI, Zhejiang, China). The sections were fixed with iced acetone and blocked by 5% BSA. The sections were incubated with the primary antibody rabbit anti-CD31 (1:100, P16284, affinity, Changzhou, China) overnight at 4°C, and the second fluorescent antibody Cy3–conjugated Goat Anti-Rabbit IgG (H + L) (1:100, SA00009-2, Proteintech™, Wuhan, China). 4’,6-diamidino-2-phenylindole (DAPI) labeled nuclei. Images were photographed by a fluorescent microscope (Carl Zeiss, Axio Scope A1, Germany) and processed by ZEN blue 2.3 software (Carl Zeiss, Germany). The circumference of the vessel’s inner wall and the CD31 positive extent were calculated by Image J software (NIH, Littleton, CO, USA).

### 5-Bromodeoxyuridinc (BrdU) incorporation assay

BrdU incorporation assay was performed as described previously [21]. EPCs were seeded on coverslips in six-well plates with LG and HG medium to 60–70% confluences and then incubated with serum-deprived LG and HG medium for 12 h. EPCs were labeled with BrdU (10 μΜ, Solarbio, Beijing, China) for 2 h. After being fixed by 4% PFA, perforated by 0.3% Triton X-100 and denatured by 2 M HCl, EPCs were incubated with mouse anti-BrdU primary antibody (1:100, Proteintech™, Wuhan, China) overnight, followed by Cy3–conjugated Goat Anti-mouse IgG (H + L) (1:100, Proteintech™, Wuhan, China), nuclei were labeled by DAPI. The immunofluorescence images were taken by a Fluorescence microscope (Carl Zeiss, scope A1, Germany). The percentage of BrdU positive cells to the total amount of cells was calculated by Image J software (NIH, Littleton, CO, USA).

### Advanced glycation end products (AGEs), hydrogen peroxide, and SA-β-gal detection

According to our report [19]. The abundance of AGEs in EPC supernatant and mice serum was detected by using a commercial kit (ab273298, Abcam, USA). Hydrogen Peroxide content in EPC was detected by using a commercial kit (Solarbio, BC3595, Beijing, China). Accumulation of Senescence-Associated β-galactosidase (SA-β-gal) in EPC was detected by using a commercial kit (C0602, Beyotime, Shanghai, China).

### Statistics

Statistical analysis was performed with IBM SPSS Statistics 20 software, according to our previous study [18]. Data are presented as means ± SE. Significant differences between and within multiple groups were examined by using ANOVA for repeated measures, followed by Duncan’s multiple range test. The Independent-Samples t-test was used to detect significant differences between the two groups. *P* < 0.05 was considered statistically significant.

## Results

### STS inhibited the NLRP3 inflammasome activation and dysfunctions of EPC in HG

We found that the expression of NLRP3 and the degree of procaspase-1 converting to caspase-1 and proIL-1β converting to IL-1β were increased by HG, which was similar to the NLRP3 inflammasome positive control, LPS + ATP treatment (Fig. 1a, b). Furthermore, immunofluorescence results showed that HG and LPS + ATP treatments significantly merged NLRP3 (red fluorescence dots) and ASC (green fluorescence dots) to form yellow dots, representing NLRP3 inflammasome assembly (Fig. 1c, d).

STS inhibited the expression of NLRP3, the production of p20 and IL-1β, and the colocalization of NLRP3 and ASC (Fig. 1a-d). Thus, HG caused significant activation of NLRP3 inflammasome in EPC; STS inhibited the activation.

Similar to the LPS + ATP treatment, HG significantly increased the expression of DNA damage index γ-H2AX (Fig. 1e-h), the expression of cell senescence-related index p21 (Fig. 1e, f), and the SA-β-gal positive area (Fig. 1i, j). At the same time, HG caused a decrease in the Ki67 (a cell proliferation index) (Fig. 1e, f) and in EPC differentiation capability as shown by CD31 and vWF expression (Fig. 1e, f). STS treatment alleviated the above EPC dysfunctions caused by HG and LPS + ATP (Fig. 1e-j).

These results indicate that STS alleviates the DNA damage of HG-induced EPC, down-regulated proliferation, differentiation, and accelerated senescence while inhibiting the HG-induced activation of EPC NLRP3 inflammasome.

### STS ameliorated the functions of EPC by inhibiting NLRP3 inflammasome-mediated catalase (CAT) inactivation in HG

**Fig. 2:**
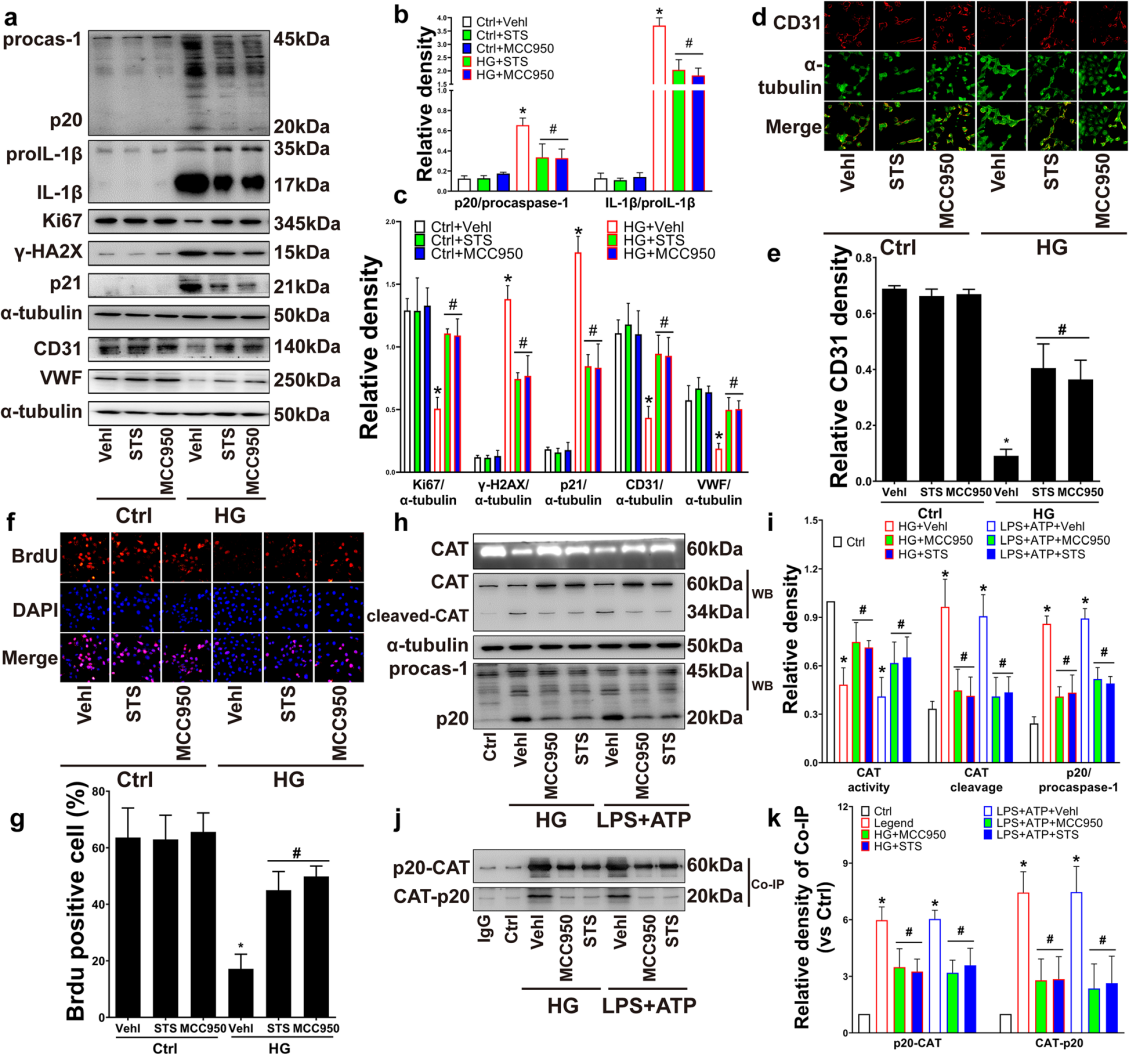
STS ameliorated HG-impaired EPC functions by inhibiting NLRP3 inflammasome activation mediated catalase (CAT) cleavage. **a** Representative Western blot gel and summarized data show the p20 and IL-1β production, representative Western blot gels show the protein of Ki67, γ-H2AX, p21, CD31, VWF expression; **b**, **c** Summarized data show the production of p20, IL-1β, Ki67, γ-H2AX, p21, CD31, VWF expression; **d** Representative images of CD31 on cell cytomembrane (200 ×), α-tubulin as control, and **e** summarized relative CD31 density; **f**, **g** Representative immunofluorescence images of BrdU incorporation (200 ×) and summarized percent of BrdU positive area in the nucleus; **h**, **i** Representative PAGE gel and summarized data show the CAT activity, representative Western blot gel and summarized data show and the degree of CAT cleavage and p20 production; **j**, **k** Representative co-immunoprecipitation gel and summarized data show the p20-CAT and CAT-p20 coprecipitation. *P < 0.05 vs. Control (Ctrl); #*P* < 0.05 vs. HG treated group or LPS + ATP treated group (*n* = 3)

To investigate the potential mechanism of the functional impairment of EPC, EPCs were treated with NLRP3 inflammasome-specific inhibitor MCC950 [22]. Similar to MCC950, STS significantly inhibited the production of inflammasome activation markers active caspase-1 (p20) and mature IL-1β (Fig. 2a, b), inhibited the expression of γ-H2AX (Fig. 2a, c), and restored the expression of Ki67 (Fig. 2a, c) and the percentage of BrdU incorporation positive cells (Fig. 2f, g); the expression of CD31, vWF (Fig. 2a, c) and the positive area of CD31 immunofluorescence (Fig. 2d, e). These results indicated that STS improved the dysfunctions of EPC by inhibiting the activity of the NLRP3 inflammasome in HG.

More, along with the decreased p20 production, STS inhibited the fragment production of cleaved CAT (a 34 kDa fragment) and restored the impaired CAT activity (Fig. 2h, i). We further found an interaction between caspase-1 (p20) and CAT by Co-IP (Fig. 2j, k).

These results indicate that STS improves the dysfunctions of EPC by inhibiting the NLRP3 inflammasome-mediated CAT inactivation in HG.

### STS alleviated the dysfunctions of EPC by inhibiting caspase-1-dependent catalase inactivation in HG

**Fig. 3:**
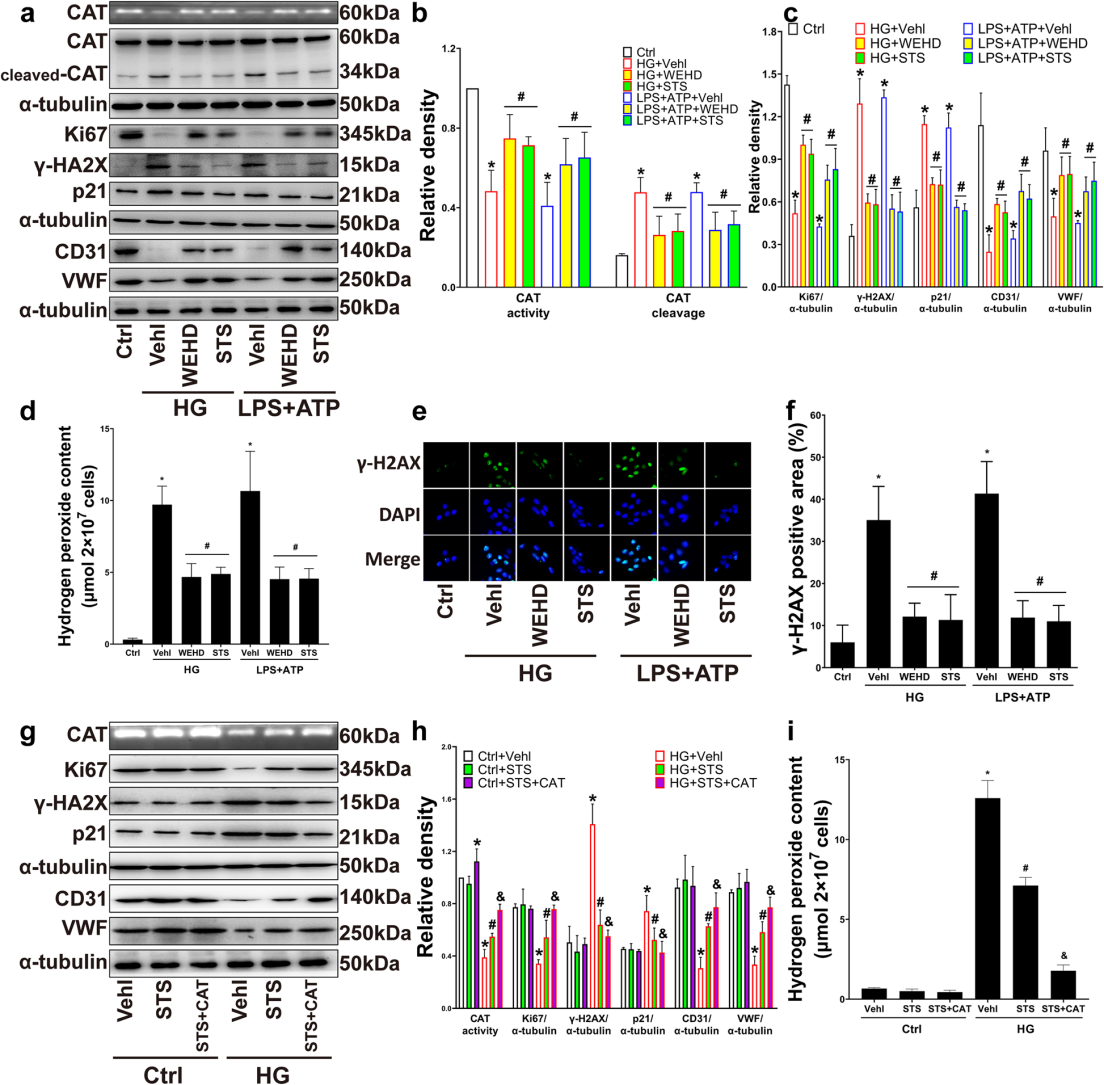
STS alleviated EPC dysfunctions by inhibiting caspase-1-dependent catalase inactivation. **a**–**c** Representative PAGE gel and summarized data show the CAT activity, representative Western blot gel and summarized data show the degree of CAT cleavage; representative Western blot gel and summarized data show the protein of Ki67, γ-H2AX, p21, CD31, VWF expression; **d** Summarized data show hydrogen peroxide accumulation in WEHD or STS treated EPC; **e**, **f** Representative immunofluorescence images of γ-H2AX in the nucleus (200 ×) and summarized data show percent of the γ-H2AX positive area in nucleus; **g**, **h** Representative PAGE gel and summarized data show the CAT activity, representative Western blot gel and summarized data show the protein of Ki67, γ-H2AX, p21, CD31, VWF expression; **i** Summarized data show hydrogen peroxide accumulation in PEG-CAT or STS treated EPC. *P < 0.05 vs. Control (Ctrl); #*P* < 0.05 vs. HG or LPS + ATP treated group; & *P* < 0.05 vs. HG + STS group (*n* = 3)

The above results concluded that STS inhibited NLRP3 inflammasome activity and improved the damage of EPC by protecting CAT; we found that active caspase-1 interacted with CAT. Therefore, EPCs were treated with a caspase-1 inhibitor WEHD [23]. The results showed that similar to WEHD, STS restored the activity of CAT, reduced the production of cleaved CAT (Fig. 3a, b), and inhibited the accumulation of hydrogen peroxide in HG (Fig. 3d). STS decreased the expression of γ-H2AX and the percentage of its immunofluorescence positive area (Fig. 3a, c, e, f); STS also reduced the expression of p21 and restored the expression of Ki67, CD31, and vWF (Fig. 3a, c).

In addition, the effects of STS on inhibiting the expression of γ-H2AX and p21, restoring the expression of Ki67, CD31, and vWF, inhibiting the accumulation of hydrogen peroxide were enhanced by exogenous CAT (Fig. 3g-i).

These results indicate that caspase-1 activation weakens CAT activity and leads to EPC DNA damage and dysfunctions. STS reverses the damage by protecting CAT activity from the HG-induced caspase-1 activation.

### STS prevented caspase-1 mediated CAT inactivation and EPC dysfunctions by inhibiting the AGE-RAGE-TXNIP pathway

When stimulated by HG or S-adenosylhomocysteine (SAH), TXNIP breaks away from the connection with thioredoxin (TRX), interacts with NLRP3 molecules, and activates NLRP3 inflammasome, causing the release of IL-1β [24]. AGEs are heterogeneous molecules derived from non-enzymatic products of carbohydrate and protein or lipid reactions [25], RAGE is a transmembrane immunoglobulin that could be activated by AGE. Under normal and healthy conditions, RAGE is expressed at the basic level. However, in pathological conditions or chronic inflammation, such as diabetes (DM), cardiovascular disease, Alzheimer’s disease, cancer and natural aging, the expression of RAGE is up-regulated. AGE-RAGE pathway is believed to be related to the activation of NLRP3 inflammasome in diabetes [26].

**Fig. 4:**
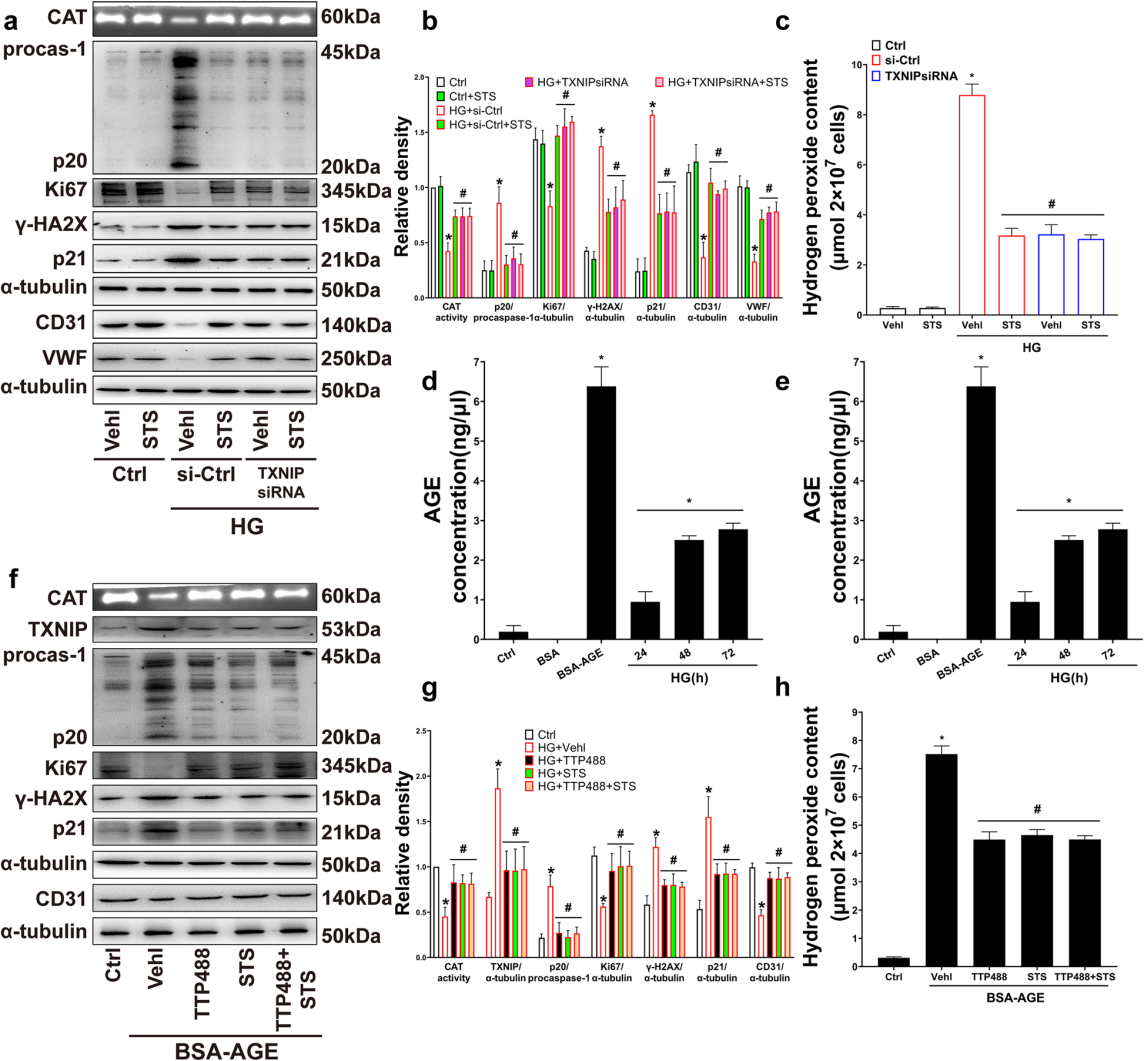
STS prevented caspase-1 mediated CAT inactivation and EPC dysfunctions by inhibiting the AGE-RAGE-TXNIP pathway. **a**, **b** Representative PAGE gel and summarized data show the CAT activity, representative Western blot gel and summarized data show the p20 production and the protein of Ki67, γ-H2AX, p21, CD31, VWF expression; **c** Summarized data show hydrogen peroxide accumulation in TXNIP siRNA treated EPC; **d**, **e** Summarized data show AGE content in EPC culture supernatant; **f**, **g** Representative PAGE gel and summarized data show the CAT activity, representative Western blot gel and summarized data show the p20 production and the protein of TXNIP, Ki67, γ-H2AX, p21, CD31, VWF expression; **h** Summarized data show hydrogen peroxide accumulation in TTP488 treated EPC. *P < 0.05 vs. Control (Ctrl); #*P* < 0.05 vs. HG or BSA-AGE treated group (*n* = 3)

EPCs were treated with TXNIP siRNA. TXNIP gene silencing significantly inhibited the activation of caspase-1, the damage of CAT activity (Fig. 4a, b), the accumulation of hydrogen peroxide (Fig. 4c), the expression of γ-H2AX and p21 (Fig. 4a, b), and restored the expression of Ki67, CD31, and vWF (Fig. 4a, b). STS had a similar effect as TXNIP siRNA (Fig. 4a-c). These results indicated that STS restored the activity of CAT, the proliferation, and differentiation functions of EPC and inhibited DNA damage and the senescence of EPC by inhibiting the TXNIP expression.

With increased HG treatment time and concentration, AGEs accumulated in the EPC culture medium (Fig. 4d, e). These results indicated that the excess glucose in the culture medium induced the formation of AGE. EPCs were treated with BSA-AGE (ab273298, Abcam, USA), and the RAGE pathway was blocked by TTP488, a small-molecule inhibitor of RAGE [27]. TTP488 inhibited the expression of TXNIP, the activation of caspase-1, the damage of CAT activity (Fig. 4f, g), the accumulation of hydrogen peroxide (Fig. 4h), and the expression of γ-H2AX and p21 (Fig. 4f, g), and also restored the expression of Ki67 and CD31 (Fig. 4f, g). STS had a similar effect as TTP488 (Fig. 4f-h).

These results indicate that excess glucose activates the RAGE-TXNIP pathway by forming AGE products to induce active caspase-1 mediated CAT activity damage, resulting in the accumulation of hydrogen peroxide and the damage of DNA, and ultimately impaired the proliferation and differentiation and accelerated senescence, STS blocks the RAGE-TXNIP pathway to protect CAT activity in EPC.

### STS protected the repairment of endothelium mediated by EPCs transplantation and prevented the aggravated neointima hyperplasia in diabetic mice

**Fig. 5:**
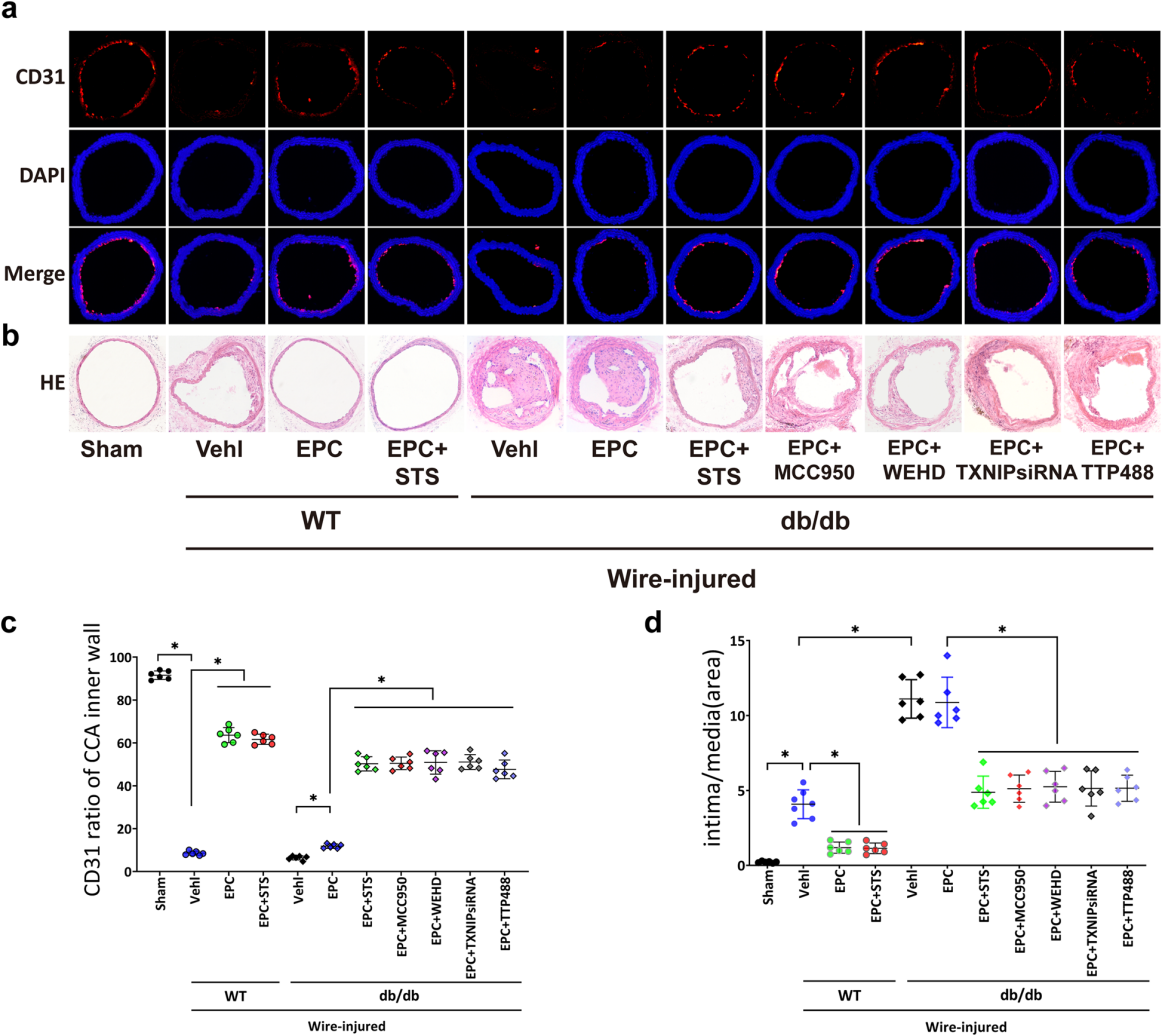
STS protected EPC-mediated endothelium repair and neointima prevention in diabetic mice. **a** Representative immunofluorescence images (100 ×) of CD31 expression in CCA inner wall and **b** Representative images (100 ×) of neointima in CCA with or without EPCs transplantation; **c** Summarized data show CD31 expression ratio of CCA inner wall; **d** Summarized data show the area ratio of intima/media. *P < 0.05 (*n* = 6)

We transplanted EPCs into WT mice and db/db mice and found that EPC transplantation promoted the expression of CD31 in WT mice but disabled in diabetic mice (Fig. 5a, c). EPCs transplantation reduced the neointima area but this did not occur in diabetic mice (Fig. 5b, d). STS treatment significantly restored the expression of CD31 in the vascular inner wall and reduced the neointima hyperplasia after EPCs transplantation (Fig. 5a-d). STS effects on the expression of CD31 and neointima reduction after EPCs transplantation were similar to the MCC950, WEHD, TXNIP siRNA, and TTP488 treatment (Fig. 5a-d).

These results suggested that the repairment of endothelium and the prevention of neointima hyperplasia of EPCs transplantation were disabled in the diabetic environment; STS restored these functions, and the mechanism was associated with the inhibition of RAGE, TXNIP, NLRP3 inflammasome, caspase-1 pathways in EPC.

## Discussion

As the core product of the NLRP3 inflammasome assembly, caspase-1 has specific aspartic-cysteine protease activity and its most widely known role is to mediate the maturation of the pro-inflammatory cytokines IL-1β and IL-18, and the pyroptosis protein Gasdermin-D [28]. Furthermore, according to the previous studies, more than 120 substrates of caspase-1 were found; they affected the cytoskeleton structure, programmed cell death, glucose and lipid metabolism, autophagy flow, and even cell differentiation and other cellular functions [29–33]. The results presented in this study showed that active caspase-1 cleaved CAT to produce a fragment of about 34 kDa, along with CAT activity decrease, leading to the accumulation of hydrogen peroxide, the damage of DNA, and eventually the dysfunctions of EPC. Previous findings seemed to provide some potential correlation between the pathway of NLRP3 inflammasome-caspase-1 and the activity of CAT. CAT activity tends to decrease with activation of NLRP3 inflammasomes [34–36], and inhibiting NLRP3 inflammasome plays a role in restoring CAT activity [37]. Our results demonstrated that caspase-1 interacted with CAT, cleaved, and significantly inactivated it, and might explain one of the mechanisms by which HG damages CAT activity. However, the mechanism of caspase-1 interaction with CAT still needs further study. STS has been reported to enhance CAT activity and the protein level of CAT in human neuroblastoma cells [38]. Our results further revealed that STS protects CAT activity by inhibiting the cleavage and inactivation of CAT mediated by HG-induced caspase-1. This conclusion provides evidence that suggests a novel antioxidant mechanism of STS.

Recent studies have found that RAGE triggers a respiratory inflammatory response through the activation of RAGE/high mobility group box-1 (HMGB1) signaling pathway [39]; meanwhile, excess glucose in the HG medium reacted with protein in serum to generate AGE, which caused inflammatory diabetic cardiomyopathy [40]. These conclusions indicate that the HG-AGE-RAGE pathway is vital in activating NLRP3 inflammasomes. Our results supported this inference and concluded that excessive glucose in the EPC medium formed AGE products, triggering RAGE and activating the NLRP3 inflammasome. Studies also concluded that TXNIP interacted with the NLRP3 molecule and caused the activation of the NLRP3 inflammasome [41]. Our results suggest that STS inhibits the activity of NLRP3 inflammasome through the suppression of TXNIP expression, which is similar to the conclusion that STS protects against ischemia/reperfusion myocardial injury by inhibiting the TXNIP over-expression [42]. We concluded that STS inhibited the RAGE-TXNIP pathway, but the mechanism of RAGE inhibition needs further investigation.

Our study demonstrated that STS protects EPC functions by inhibiting the RAGE-TXNIP-NLRP3 inflammasome pathway, which provides a new mechanism of EPC NLRP3 inflammasome activation in HG condition.

## Conclusion

STS protects the activity of CAT and the functions of EPC from the HG-induced RAGE-TXNIP-NLRP3 inflammasome pathway activation. STS restores the endothelium repairment mediated by EPCs transplantation and prevents the aggravated neointima hyperplasia in diabetic mice. Our conclusion provides a new target to protect EPC functions in diabetes and provides evidence for novel clinical applications of STS.

### Supplementary Information

**Additional file 1: Supplemental Figure 1.** Characterization of Bone Marrow-Derived EPCs.

**Additional file 2.**

## Data Availability

The datasets used and analyzed in the current study are available from the corresponding author based on reasonable request.

## Abbreviations

EPCs: Endothelial Progenitor Cells
STS: Sodium Tanshinone IIA Sulfonate
HG: High Glucose
AGE: Advanced Glycation End Product
RAGE: Receptor of Advanced Glycation End Products
CCA: Common Carotid Artery
CAT: Catalase
MNCs: Mononuclear Cells
HE: Hematoxylin-Eosin staining
Co-IP: Co-Immunoprecipitation
SA-β-gal: Senescence-Associated β-galactosidase
WEHD: Z-WEHD-FMK
VCAM-1: Vascular Cell Adhesion Molecule 1
ICAM-1: Intercellular Adhesion Molecule-1
