# Supplementary figures and images for: Sodium tanshinone IIA sulfonate ameliorates neointima by protecting endothelial progenitor cells in diabetic mice
Source: BMC Cardiovasc Disord. 2023 Sep 11;23:446. doi: 10.1186/s12872-023-03485-4 (PMC10494373; doi:10.1186/s12872-023-03485-4)

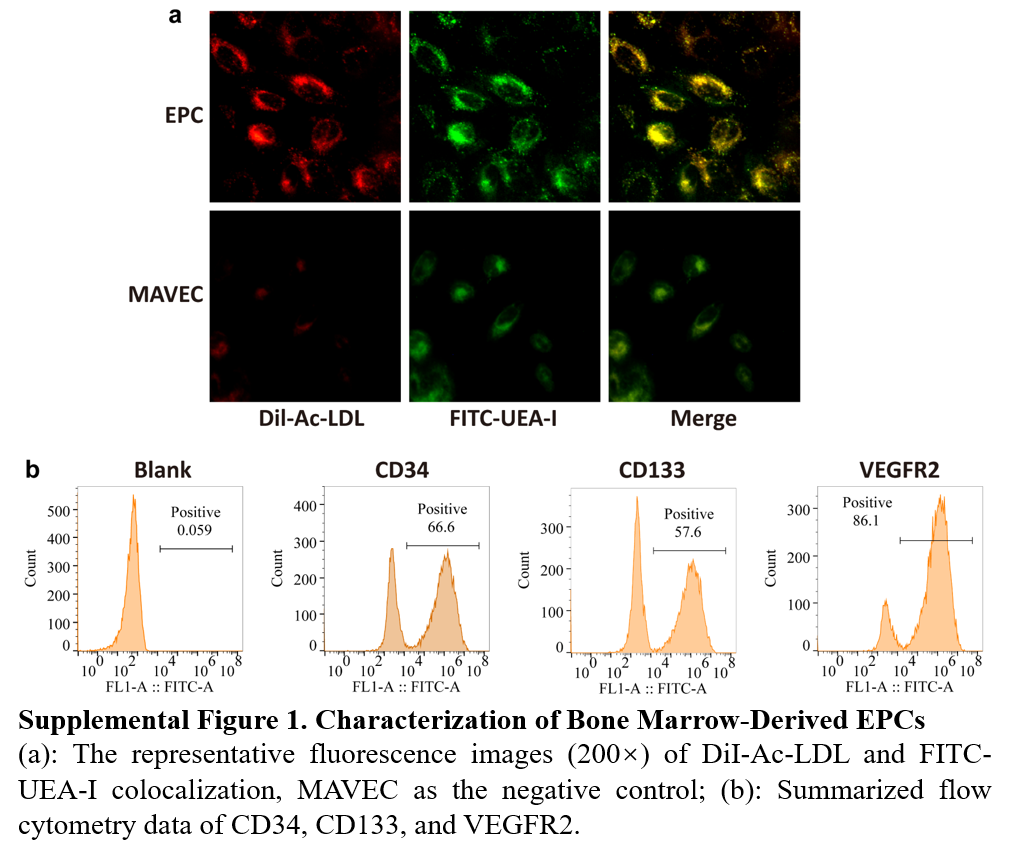

Supplement: Supplementary file 1 — Additional file 1: Supplemental Figure 1. Characterization of Bone Marrow-Derived EPCs. [file 12872_2023_3485_MOESM1_ESM.tif]

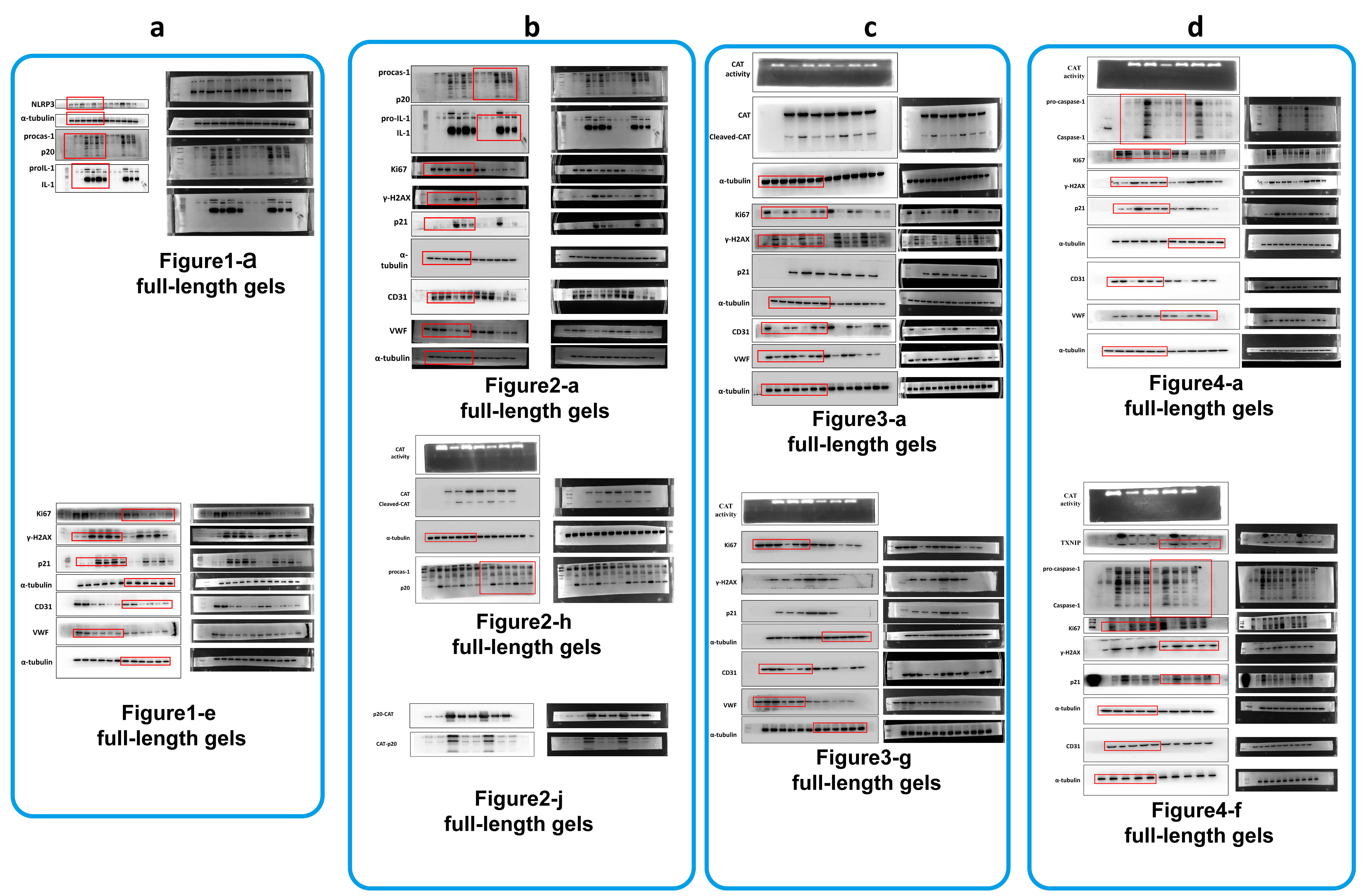

Supplement: Supplementary file 2 — Additional file 2. [file 12872_2023_3485_MOESM2_ESM.tif]
